# From the completion of neoadjuvant chemotherapy to surgery for colorectal cancer liver metastasis: What is the optimal timing?

**DOI:** 10.1002/cam4.3283

**Published:** 2020-09-04

**Authors:** Qichen Chen, Rui Mao, Jianjun Zhao, Xinyu Bi, Zhiyu Li, Zhen Huang, Yefan Zhang, Jianguo Zhou, Hong Zhao, Jianqiang Cai

**Affiliations:** ^1^ Department of Hepatobiliary Surgery National Cancer Center/National Clinical Research Center for Cancer/Cancer Hospital, Chinese Academy of Medical Sciences and Peking Union Medical College Beijing China

**Keywords:** colorectal cancer liver metastasis, neoadjuvant chemotherapy, outcomes, time to surgery

## Abstract

**Background:**

Neoadjuvant chemotherapy (NAC) has been widely performed in the treatment of colorectal cancer liver metastasis (CRLM) patients, but the optimal timing of surgery after NAC is unclear. The aim of this study was to investigate the optimal timing of surgery.

**Methods:**

From December 2010 to May 2018, 101 consecutive patients who received NAC followed by liver resection for CRLM were included in this study. The main outcome parameters were pathological response, progression‐free survival (PFS), and overall survival (OS). The effect of time to surgery (TTS) on patient outcomes, defined as a high TTS and a low TTS according to an X‐tile analysis, was investigated. To adjust for potential selection bias, propensity score matching at 1:2 was performed with two high TTS patients matched to one low TTS patient. Kaplan‐Meier curves, logistic regression analyses, and Cox regression models were used for the data analysis.

**Results:**

The optimal cut‐off value for the TTS was 5 weeks by X‐tile analysis. The patients in this study were divided into low (≤5 weeks, n = 27) and high (>5 weeks, n = 74) TTS groups. Patients with a high TTS were more likely to have an unfavorable pathological response (75.7% vs 48.1%, *P* = .008). In multivariate analysis, a low TTS significantly predicted a better pathological response (OR = 3.397, 95% CI: 1.116‐10.344, *P* = .031). Compared to patients with a high TTS, patients with a low TTS had significantly better PFS (*P *<* *.001, mPFS: 16 months vs 7 months) and better OS (*P* = .037, mOS: not reached vs 36 months). Multivariate analysis revealed that a TTS > 5 weeks was an independent predictor of decreased PFS (HR = 2.041, 95% CI: 1.152‐3.616, *P* = .014) but not OS. After propensity matching, the patients with a low TTS had significantly better PFS (*P *< .001, mPFS: 18.2 months vs 10 months) and an equivalent OS (*P* = .115, mOS: not reached vs 41 months). Multivariate analysis revealed that a TTS > 5 weeks was an independent predictor of decreased PFS (HR = 3.031, 95% CI: 1.494‐6.149, *P* = .002) but not OS.

**Conclusion:**

The longer TTS after the completion of NAC may be disadvantageous for a favorable pathological response and long‐term PFS. These results should be validated prospectively in a randomized trial.

## Graphical abstract

An interval of 5 weeks between the completion of NAC and surgical resection for CRLM is an inflection point in pathological response and survival. The cohort of patients receiving resection between 4 and 5 weeks after NAC demonstrated a higher rate of favourable pathological response and better PFS. This finding was confirmed with a secondary propensity score matching analysis.

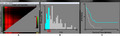

## 1 INTRODUCTION

Neoadjuvant chemotherapy (NAC) followed by curative resection has been increasingly advocated to prolong the survival of patients with potential resectable colorectal cancer liver metastasis (CRLM), as NAC reduces micrometastases, downstages the tumor, and improves the tumor resection rate,^1^, ^2^ although NAC has some potential disadvantages: the risk of progression of tumor, the local fibrosis and tissue adhesion caused by NAC and the damage of NAC toxicity (sinusoidal obstruction syndrome, nodular regenerative hyperplasia and hematologic toxicities, et al) to body function.^3^, ^4^, ^5^ Surgeons are frequently confronted with the question of scheduling surgery at an appropriate time after the completion of NAC for CRLM patients. The current clinical guidelines^6^, ^7^ recommend scheduling CRLM resection after 4 weeks from the last dose of NAC. However, the optimal timing for CRLM resection after 4 weeks has still not been defined.

The choice of the interval between the completion of neoadjuvant therapy and surgery was determined by several factors, including the prolonged effect of neoadjuvant therapy, the physical and nutritional status impacted by comorbidities after neoadjuvant therapy and the risk of tumor progression. A longer time to surgery (TTS) may potentially allow the tumor to continue to regress because of a prolonged effect of neoadjuvant therapy. However, the risk of primary or metastatic tumor regrowth is increasing. Some studies have indicated that extending TTS might increase the proportion of patients with a pathologic complete response (pCR) among rectal cancer patients receiving neoadjuvant chemoradiotherapy (nCRT).^8^, ^9^ In addition, for esophageal cancer patients with nCRT and gastric cancer patients with NAC, studies revealed that patients with a longer TTS had significantly higher pCR rates and better prognosis.^10^, ^11^, ^12^ However, other studies have not shown the relationship between longer TTS and higher pCR rates and have revealed that longer TTS was associated with worse overall survival.^13^, ^14^ For CRLM, the impact of TTS after the completion of NAC on pathological response and survival is still not reported.

This study is the first to investigate the optimal timing of surgery for CRLM patients. The purpose of this study was to identify the specific timing associated with an inflection point in clinical outcome and compare the clinical outcomes before and after this specific timing.

## 2 MATERIALS AND METHODS

### 2.1 Patients and therapy

**FIGURE 1:**
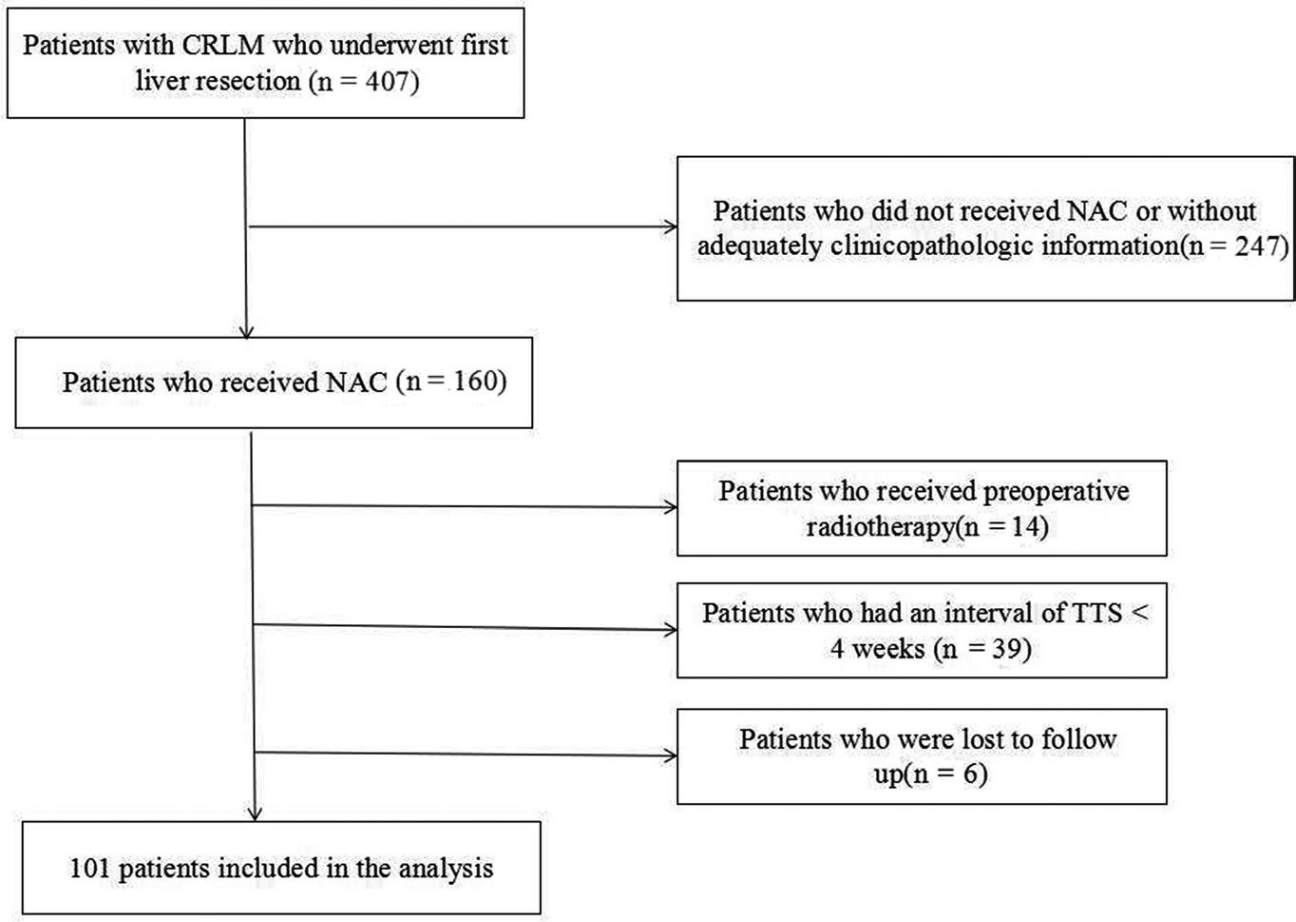
Flow diagram for the selection of colorectal cancer liver metastasis (CRLM) included in the final analyses of this study.

From December 2010 to May 2018, 101 consecutive patients who received NAC followed by first liver resection for CRLM at our hospital were included in this study. Patients who received preoperative radiotherapy, had an interval of TTS < 4 weeks after the completion of NAC, or underwent palliative resection surgery (R2 resection) were excluded. Flow diagram for the selection of CRLM included in the final analyses of this study is shown in Figure 1. Ethical approval was obtained from the Institutional Review Board of the Cancer Hospital, Chinese Academy of Medical Sciences.

**FIGURE 2:**
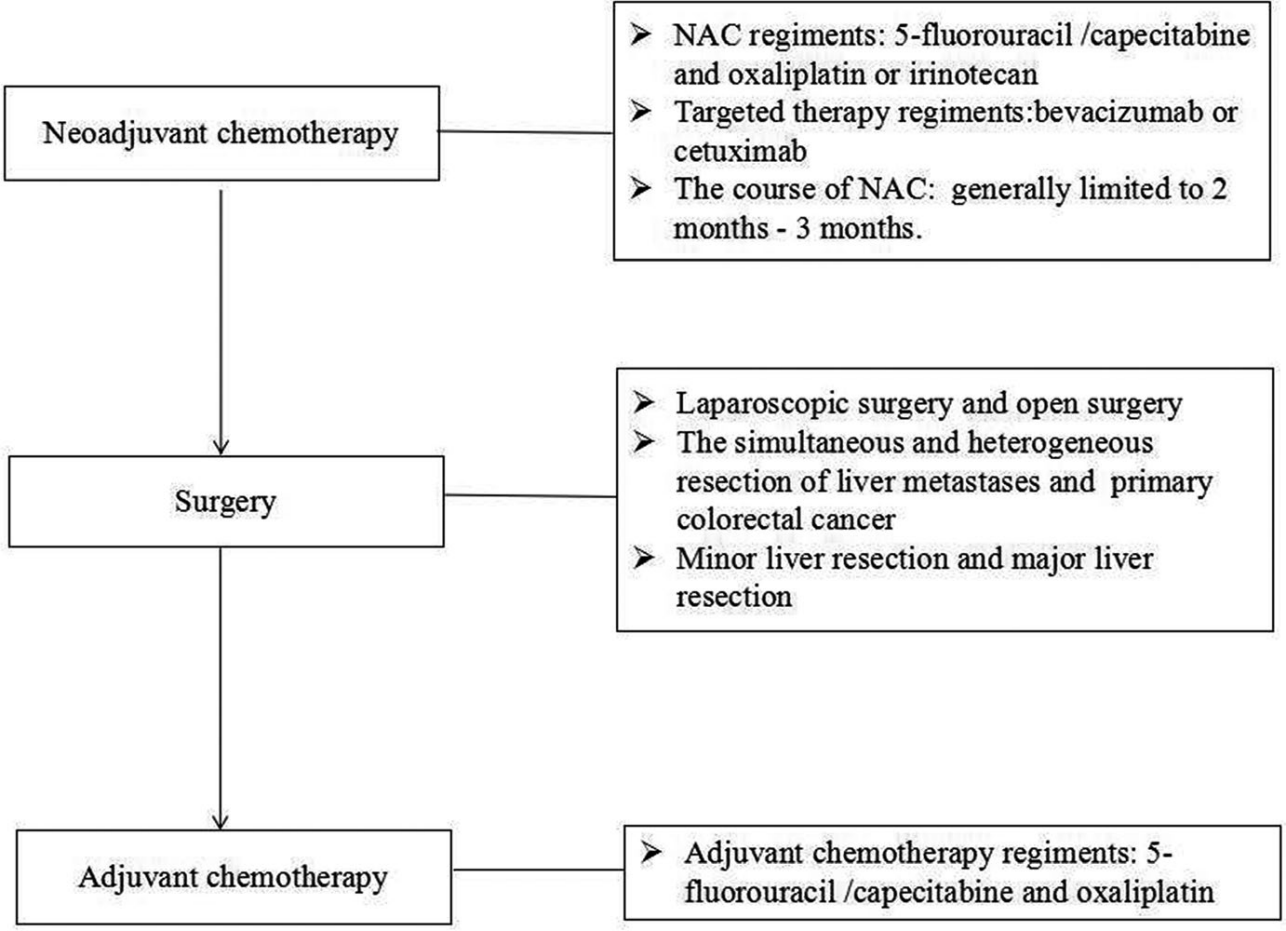
Flowchart about the treatment strategy of colorectal cancer liver metastasis patients in this study.

The treatment strategies for CRLM were determined by a multidisciplinary team as described previously.^5^, ^15^ A flowchart about the treatment strategy of CRLM patients in this study is shown in Figure 2. All patients in this study were evaluated with respect to potential resectability by sophisticated surgeons before the administration of NAC. NAC was recommended to CRLM patients with high clinical risk‐scoring systems score^16^, ^17^, ^18^ or initially unresectable liver metastases, which was consistent to the criteria of NAC in guidelines.^7^, ^19^ The regimens of NAC consisted of 5‐fluorouracil/capecitabine and oxaliplatin or irinotecan. NAC toxicity was graded according to the NCI‐CTCAE (version 4.0).^20^ According to RECIST criteria,^21^ the clinical response to NAC was evaluated. A complete response or a partial response was defined as a favorable clinical response. The pathological response was evaluated according to tumor regression grade (TRG).^22^ Pathological TRG 1‐3 was described as a favorable response to NAC.

When the CRLM was resectable, liver resection was scheduled at least 4 weeks after NAC. Patients received adjuvant chemotherapy according to the histological stage and the pathological response. For patient with synchronous CRLM, both primary and metastatic tumors were resected simultaneously. The comorbidities were defined as chronic medical diseases such as diabetes, hypertension, cardiac disease, and so on. The Clavien‐Dindo classification system was used to describe the severity of each postoperative complication, and major complications were classified as Clavien‐Dindo Ⅲ‐Ⅴ.^23^ Liver resections were defined as major or minor resections. Resections of one segment were defined as minor liver resections. Preoperative performance status of CRLM patients was evaluated by controlling nutritional status (CONUT) score, BMI, and albumin levels. The CONUT score was divided into low CONUT (<2) and high CONUT (≥2). The high CONUT represented the poor performance status.^24^, ^25^

### 2.2 Follow‐up and outcome

All patients were required to visit the clinics every 3 months during the first 2 postoperative years, every 6 months thereafter for 3 years, and yearly after 5 years. Contrast‐enhanced CT or MRI scan was routinely implemented every 6‐12 months. After recurrence, the patients received liver resection, radiofrequency, or chemotherapy. Overall survival (OS) was defined as the interval between the date of liver resection and the date of death or the last follow‐up, and progression‐free survival (PFS) was defined as the duration from liver resection to tumor progression or the last follow‐up.

### 2.3 Statistical analysis

The chi‐square test or Fisher's exact test was performed to analyze the distribution of categorical data. Continuous data were analyzed by the Mann‐Whitney *U* test. Survival was calculated with the Kaplan‐Meier method and compared by a log‐rank test. All predictors with *P* < .10 by univariate analysis were retained in multivariate models. Multivariate analysis using the Cox proportional hazards regression analysis was performed to investigate independent factors of survival. Forward LR was used in the multivariate analysis. For survival, an X‐tile analysis^26^ was implemented to investigate the optimal cut‐off values for the TTS. Owing to differences between the high TTS group and the low TTS group in terms of the baseline characteristics, a 1:2 propensity score matched analysis was used to adjust for these differences. Statistical significance was set at two‐sided *P* < .05. Statistical analyses were performed by SPSS, version 22 software.

## 3 RESULTS

### 3.1 Clinicopathologic characteristics

A total of 101 patients in our study consisted of 63 males and 38 females and the median age was 56 years (IQR 50‐63). The range of TTS was 28‐83 days and the median TTS was 41 days (IQR 35.0‐51.0). The median albumin level was 42 g/L (IQR 39.2‐44.0). There were 27 patients with high CONUT score and BMI > 24 kg/m^2^ was observed in 53 patients. Fifty patients (49.5%) had primary tumors located in the rectum. The pT3‐pT4 stage was observed in 88.1% of the patients. A node‐positive primary tumor was found in 69.3% of the patients. The pN1 stage was observed in 47.5% of the patients and the pN2 stage was observed in 21.8% of the patients. There were 88 patients (87.1%) with synchronous CRLM. The median diameter of the largest liver metastasis was 2.5 cm (IQR 1.6‐3.9), and 47 patients had a liver metastasis >3 cm. Of these patients, 73.3% had more than one liver metastasis, with a median of three lesions (IQR 1.0‐5.0).

Eighty‐eight patients (87.1%) received an oxaliplatin‐based regimen. Thirty‐nine patients (38.6%) underwent targeted therapy. Nineteen patients underwent bevacizumab therapy and 20 patients received cetuximab therapy. Twenty‐four patients (23.8%) received more than seven NAC cycles. Eighty‐four patients (83.2%) had NAC toxicities. Forty‐four patients had hematologic toxicities (grade 1‐2:35 patients; grade 3‐4: nine patients) and a total of 43 patients (43/84, 42.6%) had neutropenia. Forty‐four patients had gastrointestinal toxicity (grade 1‐2:41 patients; grade 3‐4: three patients) and nine patients held liver toxicity (grade 1:3 patients; grade 2: three patients). No mortality was observed due to NAC. A favorable histological response (TRG 1‐3) was observed in 32 patients (31.7%). The clinicopathologic characteristics of patients are shown in Table 1.

**TABLE 1:** Patient and tumor characteristics

Item	Before propensity matching				After 1:2 propensity matching			
	TTS ≤ 5 wk (n = 27)	TTS > 5 wk (n = 74)	*P*	All patients (n = 101)	TTS ≤ 5 wk (n = 24)	TTS > 5 wk (n = 37)	*P*	All patients (n = 61)
Age > 60 y, n (%)	12 (44.4)	26 (35.1)	.393	38 (37.6)	11 (45.8)	14 (37.8)	.535	25 (41.0)
Male, n (%)	14 (51.9)	49 (66.2)	.187	63 (62.4)	12 (50.0)	26 (70.3)	.111	38 (62.3)
BMI > 24 kg/m^2^, n (%)	10 (37.7)	43 (58.1)	.061	53 (52.5)	9 (37.5)	22 (59.5)	.194	31 (50.8)
ALB > 40 g/L, n (%)	16 (59.3)	51 (68.9)	.363	67 (66.3)	13 (54.2)	27 (73.0)	.131	40 (65.6)
High CONUT score, n (%)	7 (25.9)	20 (27.0)	.912	27 (26.7)	7 (29.2)	13 (35.1)	.628	20 (32.8)
Comorbidity, n (%)	10 (37.0)	38 (51.4)	.202	48 (47.5)	8 (33.3)	19 (51.4)	.166	27 (44.3)
ASA score 3‐4, n (%)	2 (7.4)	9 (12.2)	.497	11 (10.9)	3 (8.1)	2 (8.3)	.975	5 (8.2)
Preoperative CEA > 10 ng/mL, n (%)	12 (44.4)	34 (45.9)	.893	46 (45.5)	11 (45.8)	18 (48.6)	.830	29 (47.5)
Synchronous metastasis, n (%)	21 (77.8)	67 (90.5)	.090	88 (87.1)	21 (87.5)	31 (83.8)	.689	52 (85.2)
Left hemicolon, n (%)	4 (14.8)	8 (10.8)	.582	12 (11.9)	3 (12.5)	4 (10.8)	.840	7 (11.5)
R0 resection, n (%)	14 (51.9)	45 (60.8)	.419	59 (58.4)	13 (54.2)	26 (70.3)	.201	39 (63.9)
Major liver resection, n (%)	16 (59.3)	49 (66.2)	.518	65 (64.4)	13 (54.2)	25 (67.6)	.291	38 (62.3)
Heterochronous resection, n (%)	6 (22.2)	17 (23.0)	.937	23 (22.8)	5 (20.8)	10 (27.0)	.583	15 (24.6)
Bilobar distribution, n (%)	11 (40.7)	42 (56.8)	.154	53 (52.5)	9 (37.5)	20 (54.1)	.206	29 (47.5)
Extrahepatic metastases, n (%)	4 (14.8)	8 (10.8)	.582	12 (11.9)	3 (12.5)	4 (10.8)	.840	7 (11.5)
Diameter of metastases >3 cm, n (%)	13 (48.1)	34 (45.9)	.844	47 (46.5)	11 (45.8)	14 (37.8)	.535	25 (41.0)
Multiple metastases, n (%)	17 (63.0)	57 (77.0)	.158	74 (73.3)	15 (62.5)	26 (70.3)	.528	41 (67.2)
Poor differentiation, n (%)	5 (18.5)	25 (33.8)	.137	30 (29.1)	5 (20.8)	12 (32.4)	.324	17 (27.9)
pT3‐T4, n (%)	21 (77.8)	68 (91.9)	.052	89 (88.1)	18 (75.0)	32 (86.5)	.254	50 (82.0)
Node‐positive primary tumor, n (%)	19 (70.4)	51 (68.9)	.889	70 (69.3)	17 (70.8)	22 (59.5)	.366	39 (63.9)
pN1 stage, n (%)	9 (33.3)	39 (52.7)	.085	48 (47.5)	8 (33.3)	15 (40.5)	.570	23 (37.7)
pN2 stage, n (%)	10 (37.0)	12 (16.2)	.025	22 (21.8)	9 (37.5)	7 (18.9)	.107	16 (26.2)
NAC toxicity, n (%)	24 (88.9)	60 (81.1)	.353	84 (83.2)	21 (87.5)	29 (78.4)	.365	50 (82.0)
Neutropenia, n (%)	13 (48.1)	30 (40.5)	.494	43 (42.6)	11 (45.8)	16 (43.2)	.842	27 (44.3)
KRAS mutation, n (%)^a^	4 (14.8)	11 (14.9)	.679	15 (14.9)	3 (23.1)	3 (13.6)	.474	6 (17.1)
Preoperative chemotherapy								
Oxaliplatin‐based regimen, n (%)	24 (88.9)	64 (86.5)	.750	88 (87.1)	22 (91.7)	32 (86.5)	.535	54 (88.5)
Cycles > 7, n (%)	2 (7.4)	22 (29.7)	.020	24 (23.8)	2 (8.3)	6 (16.2)	.373	8 (13.1)
Targeted therapy, n (%)	11 (40.7)	28 (37.8)	.791	39 (38.6)	9 (37.5)	15 (40.5)	.812	24 (39.3)
Pathological response, n (%)	14 (51.9)	18 (24.3)	.008	32 (31.7)	12 (50.0)	15 (40.5)	.467	27 (44.3)
Clinical response, n(%)	15 (55.6)	33 (44.6)	.329	48 (47.5)	14 (58.3)	21 (56.8)	.903	35 (57.4)
Complications, n (%)	15 (55.6)	44 (59.5)	.725	59 (58.4)	13 (54.2)	21 (56.8)	.842	34 (55.7)
Minor complications, n (%)	10 (37.0)	24 (32.4)	.665	34 (33.7)	8 (33.3)	12 (32.4)	.942	20 (32.8)
Major complications, n (%)	5 (18.5)	20 (27.0)	.381	25 (24.8)	5 (20.8)	9 (24.3)	.751	14 (23.0)
Postoperative chemotherapy, n (%)	18 (66.7)	40 (54.1)	.257	58 (57.4)	17 (70.8)	20 (54.1)	.190	37 (60.7)

Abbreviations: BMI, body mass index; NAC, neoadjuvant chemotherapy; TTS, time to surgery.

^a^ KRAS status was available in 66 patients before propensity matching and in 35 patients after propensity matching.

### 3.2 Analyses of the best cut‐off point for TTS

**FIGURE 3:**
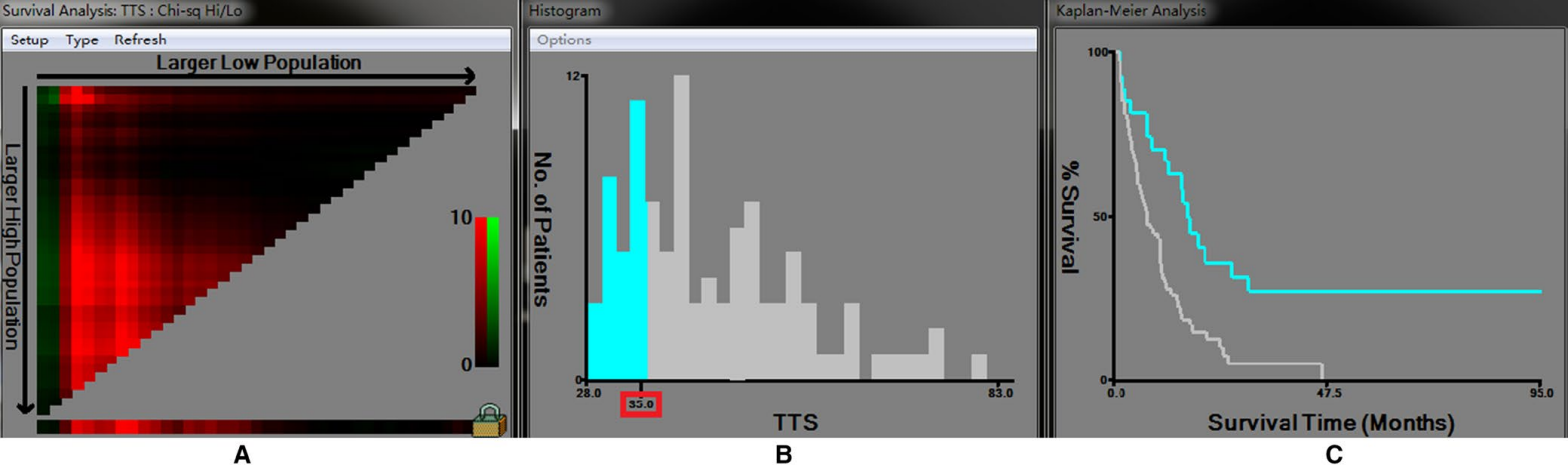
The analysis of the optimal cut‐off point for the time to surgery (TTS) by X‐tile analysis. A, The cursor can be manually moved over any coloration of the plot to choose the cut‐off point for TTS (B) to reveal survival curves (C). When the cursor was moved into the horizontal axis, the optimal cut‐off point for TTS was chosen. B, Histogram of the entire cohort divided into low TTS and high TTS subgroups according to the optimal cut‐off value of 35.0 by Figure A. Blue bars represent the low TTS group, and gray bars represent the high TTS group. C, Kaplan‐Meier plot of PFS in groups stratified using the optimal cut‐off value of TTS. Blue curves represented the low TTS group, and gray curves represented the high TTS group.

The Figure 3 shows TTS divided at the optimal cut‐point, as defined by the most significance (brightest pixel). The optimal cut‐point of TTS was 35 days and the increasing TTS was significantly associated with poor prognosis. For the analysis of the impact of TTS ≤ 5 weeks and TTS > 5 weeks on survival, please read the section of results: Impact of the TTS on survival. On the basis of the cut‐off point TTS = 5 weeks, we tried to investigate other optimal cut‐off points. The study divided TTS into three groups (4 weeks ≤ TTS ≤ 5 weeks, 5 weeks < TTS ≤ 6 weeks, TTS > 6 weeks). The results showed that patients with 4 ≤ TTS ≤ 5 weeks had better PFS and better OS than patients with 5 < TTS ≤ 6 weeks or TTS > 6 weeks. However, compared to patients with TTS > 6 weeks, patients with 5 < TTS ≤ 6 weeks did not have significantly better PFS (*P* = .943, mPFS: 7 months vs 6.7 months) and better OS (*P* = .586, mOS: 41 months vs 34 months). TTS = 6 weeks was not a proper cut‐off point. Next, we divided TTS into three groups (4 ≤ TTS ≤ 5 weeks, 5 < TTS ≤ 7 weeks, TTS > 7 weeks). The results showed that patients with 4 ≤ TTS ≤ 5 weeks had better PFS than patients with 5 < TTS ≤ 7 weeks or TTS > 7 weeks. However, compared to patients with TTS > 7 weeks, patients with 5 < TTS ≤ 7 weeks did not have significantly better PFS (*P* = .552, mPFS: 7 months vs 6.7 months). For OS analyses, patients with 4 ≤ TTS ≤ 5 weeks did not have significantly better OS than patients with 5 < TTS ≤ 7 weeks (*P* = .160, mOS: not reach vs 43.0 months). TTS = 7 weeks was not a good cut‐off point.

Based on the above analysis, the best cut‐off point was only one (TTS = 5 weeks), which was selected in this study.

### 3.3 Clinicopathologic characteristics between high TTS group and low TTS group

According to 5 weeks, the patients were divided into low (≤5 weeks, n = 27) and high (>5 weeks, n = 74) TTS groups. In the low TTS group, the range of TTS was 28‐35 days and the median TTS was 32 days (IQR 30.0‐45.0). In the high TTS group, the range of TTS was 36‐83 days and the median TTS was 47.5 days (IQR 41.0‐55.3). The median length stay after resection was 10 days (IQR 9‐13.5 days; range 6‐31 days). There was no significantly different for length stay after resection in the high TTS group (IQR 9.0‐13.3) and low TTS group (IQR 10.0‐16.0) (*P* = .374). For patient with synchronous CRLM, both primary and metastatic tumor were resected simultaneously. The range of time of NAC to colorectal cancer resection was 28‐74 days and the median time was 41 days (IQR 35.0‐50.0). In the low TTS group, the range of time of NAC to colorectal cancer resection was 28‐35 days and the median time was 32 days (IQR 30.0‐45.0). In the high TTS group, the range of time of NAC to colorectal cancer resection was 36‐74 days and the median time was 47 days (IQR 41.0‐54.5). The high TTS group has the longer timing of NAC to colorectal cancer resection than low TTS group.

There were no significant differences in nutritional status (CONUT score, BMI and albumin), comorbidity, pT stage, node‐positive primary tumor, preoperative CEA, metastasis diameter, and bilobar distribution between the low TTS group and the high TTS group. On the contrary, compared to patients with a low TTS, patients with a high TTS were more likely to receive NAC > 7 cycles (29.7% vs 7.4%) and had advanced tumor stage (pT3‐pT4 stage: 91.9% vs 77.8%). Patients with a low TTS were more likely to show a favorable pathological response (51.9% vs 24.3%). After 1:2 propensity matching, 24 patients were placed in the low TTS group, and 37 patients were placed in the high TTS group. No differences were recorded between the low TTS group and the high TTS group considering pathological T stage, chemotherapy cycle, pathological response, and lymph node invasion (all *P* values >.1). The clinicopathologic characteristics between the two groups are compared in Table 1.

### 3.4 Impact of the TTS on postoperative complications

In this study, 58.4% (59/101) of patients had postoperative complications (general complications: 42 patients; surgery‐related complications: 33 patients), including 25 major complications (25/59, 42.4%) and 34 minor complications (57.6%). There was no liver failure in this study. In the high TTS group, 44 patients had complications (general complications: 30 patients; surgery‐related complications: 26 patients), including 20 major complications and 24 minor complications. In the low TTS group, 15 patients had complications (general complications: 12 patients; surgery‐related complications: 7 patients), including 5 major complications and 10 minor complications. There were no significant differences in postoperative complications, minor complications, and major complications between the low TTS group and the high TTS group (Table 1).

### 3.5 Impact of the TTS on pathological response

The association between pathological response and clinical and pathological features are shown in Table 2. In the univariate analyses, left hemicolon (*P* = .035), TTS < 5 weeks (*P* = .008), neutropenia (*P* = .006), clinical response (*P* < .001), targeted therapy (*P* = .004), and T3‐T4 stage (*P* = .006) were associated with a favorable pathological response. Compared to patients with a low TTS, patients with a high TTS were more likely to have unfavorable pathological responses (75.7% vs 48.1%). In multivariate analysis, low TTS (OR = 3.397, 95% CI: 1.116‐10.344, *P* = .031), targeted therapy (OR = 2.959, 95% CI: 1.050‐8.336, *P* = .040), neutropenia (OR = 3.015, 95% CI: 1.077‐8.437, *P* = .036), and clinical response (OR = 5.329, 95% CI: 1.785‐15.910, *P* = .003) were independent indicators for a favorable histological response.

**TABLE 2:** Prognostic factors for the pathological response

Factor	Univariate analysis	Multivariate analysis	
	Value *P*	OR (95% CI)	Value *P*
Age > 60 y	.368		
Male	.986		
BMI > 24 kg/m^2^	.734		
ALB > 40 g/L	.918		
Comorbidity	.929		
ASA score 3‐4	.739		
High CONUT score	.830		
Preoperative CEA < 10 ng/mL	.855		
Synchronous metastasis	.176		
Left hemicolon	.035	4.399 (0.845‐22.900)	.078
Bilobar distribution	.443		
Extrahepatic metastases	.896		
Diameter of metastases ≥ 3 cm	.417		
Multiple metastases	.237		
Poor differentiation	.101		
pT3‐T4 stage	.006		
Node‐positive primary tumor	.053		
NAC toxicity	.482		
Neutropenia	.006	3.015 (1.077‐8.437)	.036
TTS ≤ 5 wk	.008	3.397 (1.116‐10.344)	.031
Preoperative chemotherapy			
Oxaliplatin‐based regimen	.475		
Cycles > 7	.191		
Targeted therapy	.004	2.959 (1.050‐8.336)	.040
Clinical response	<.001	5.329 (1.785‐15.910)	.003

Abbreviations: BMI, body mass index; NAC, neoadjuvant chemotherapy; TTS, time to surgery.

### 3.6 Impact of the TTS on survival

#### 3.6.1 Before 1:2 propensity matching

**FIGURE 4:**
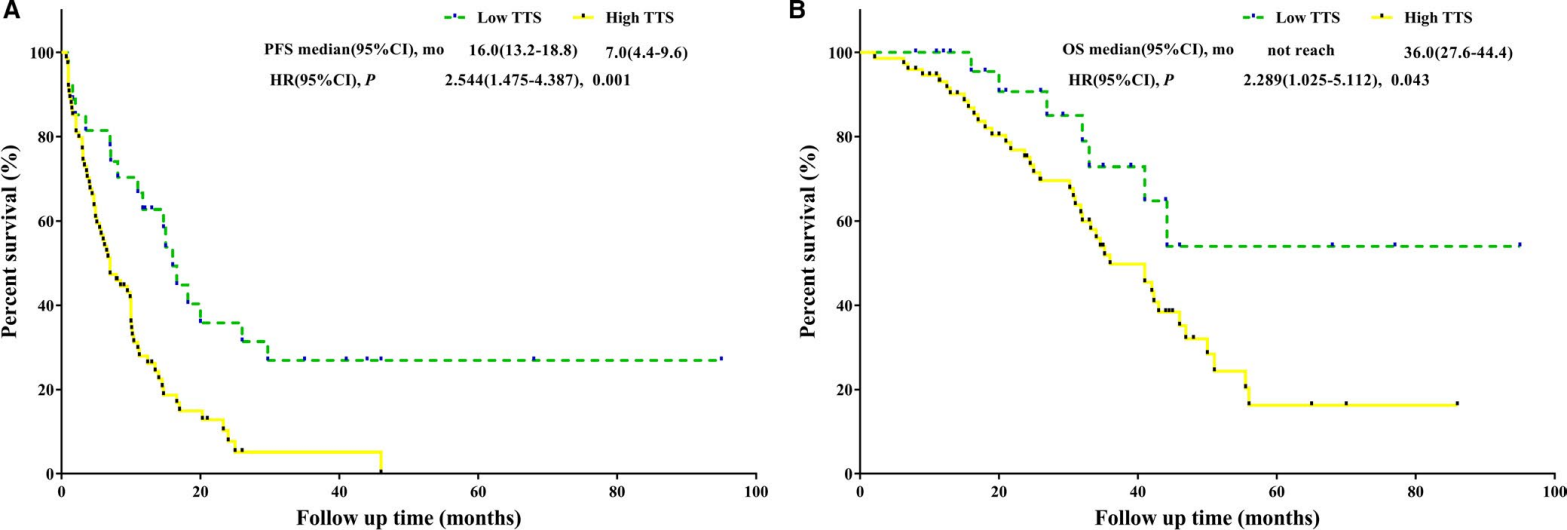
Before propensity matching. A, PFS analysis of high TTS vs low TTS. B, OS analysis of high TTS vs low TTS. OS, overall survival; PFS, progression‐free survival; TTS, time to surgery

The median follow‐up time was 44 months. Eighty‐two patients (81.19%) experienced disease recurrence, and 47 patients (46.53%) died. The median OS was 42 months (95% CI 34.3‐49.7), and the median PFS was 9.9 months (95% CI 7.2‐12.6). Compared to patients with a high TTS, patients with a low TTS had better PFS (*P *<* *.001, mPFS: 16 months vs 7 months) and better OS (*P* = .037, mOS: not reached vs 36 months) (Figure 4).

Univariate analysis revealed that age ≤ 60 years, non‐R0 resection, major liver resection, TTS > 5 weeks, T3‐T4 stage, node‐positive primary tumor, homochronous resection, nonoxaliplatin‐based regimen, >7 NAC cycles, and targeted therapy were associated with decreased PFS. Multivariate analysis revealed that TTS > 5 weeks (HR = 2.041, 95% CI: 1.152‐3.616, *P* = .014) and >7 NAC cycles (HR = 3.224, 95% CI: 1.878‐5.535, *P* < .001) were independent predictors of decreased PFS and age > 60 years (HR = 0.579, 95% CI: 0.358‐0.935, *P* = .025) and R0 resection (HR = 0.622, 95% CI: 0.401‐0.963, *P* = .033) were independent predictors of increased PFS (Table 3). The univariate analysis showed a TTS > 5 weeks was correlated with worse OS (HR = 2.289, 95% CI: 1.025‐5.112, *P* = .043). However, TTS > 5 weeks was not a significant predictor for OS in multivariate analysis (Table 3).

**TABLE 3:** Univariate and multivariate analyses of predictive factors of PFS and OS for CRLM patients before propensity matching

Factor	PFS				OS			
	Univariate analysis		Multivariate analysis		Univariate analysis		Multivariate analysis	
	Value *P*	HR (95% CI)	Value *P*	HR (95% CI)	Value *P*	HR (95% CI)	Value *P*	HR (95% CI)
Age > 60 y	.011	0.543 (0.338‐0.871)	.025	0.579 (0.358‐0.935)	.208	0.662 (0.348‐1.257)		
Male	.513	1.164 (0.739‐1.833)			.574	1.189 (0.523‐1.660)		
Preoperative CEA > 10 ng/mL	.555	1.142 (0.736‐1.771)			.811	0.932 (0.736‐1.771)		
BMI > 24 kg/m^2^	.833	1.048 (0.676‐1.625)			.592	1.170 (0.658‐2.082)		
ALB > 40 g/L	.948	0.985 (0.622‐1.560)			.385	1.313 (0.710‐2.428)		
Comorbidity	.915	0.976 (0.630‐1.513)			.825	0.937 (0.527‐1.667)		
ASA score 3‐4	.173	1.590 (0.816‐3.098)			.605	1.223 (0.570‐2.628)		
High CONUT score	.224	1.340 (0.837‐2.145)			.389	1.310 (0.708‐2.425)		
Synchronous metastasis	.548	0.828 (0.447‐1.534)			.014	0.346 (0.149‐0.806)		
Left hemicolon	.566	1.227 (0.610‐2.466)			.708	0.799 (0.246‐2.589)		
R0 resection	.017	0.588 (0.380‐0.910)	.033	0.622 (0.401‐0.963)	.016	0.484 (0.269‐0.871)	.004	0.395 (0.208‐0.749)
Major liver resection	.007	1.903 (1.192‐3.038)			.111	1.637 (0.892‐3.004)		
Bilobar distribution	.156	1.373 (0.886‐2.127)			.685	1.127 (0.663‐2.008)		
Extrahepatic metastases	.192	1.507 (0.814‐2.790)			.589	1.267 (0.537‐2.992)		
Diameter of metastases ≥ 3 cm	.056	1.531 (0.990‐2.369)			.027	1.931 (1.078‐3.459)		
Multiple metastases	.114	1.514 (0.905‐2.534)			.460	1.290 (0.656‐2.540)		
Complications	.400	1.208 (0.779‐1.873)			.018	2.006 (1.132‐3.772)		
Minor complications	.346	1.247 (0.788‐1.974)			.097	1.628 (0.915‐2.898)		
Major complications	.986	0.995 (0.594‐1.666)			.231	1.575 (0.749‐3.313)		
TTS > 5 wk	.001	2.544 (1.475‐4.387)	.014	2.041 (1.152‐3.616)	.043	2.289 (1.025‐5.112)		
Poor differentiation	.348	1.249 (0.786‐1.984)			.695	1.129 (0.615‐2.071)		
pT3‐T4	.038	2.414 (1.049‐5.553)			.204	2.137 (0.662‐6.899)		
Node‐positive primary tumor	.034	1.747 (1.042‐2.928)			.276	1.457 (0.741‐2.866)		
NAC toxicity	.147	1.577 (0.852‐2.920)			.046	4.255 (1.025‐17.655)		
Neutropenia	.877	1.035 (0.668‐1.605)			.485	1.229 (0.689‐2.195)		
Heterochronous resection	.022	1.791 (1.087‐2.950)			<.001	3.323 (1.814‐6.087)	.001	2.908 (1.527‐5.535)
Preoperative chemotherapy								
Oxaliplatin‐based regimen	.020	0.484 (0.263‐0.891)			.169	0.584 (0.271‐1.256)		
Cycles > 7	<.001	4.209 (2.491‐7.112)	.000	3.224 (1.878‐5.535)	<.001	3.360 (1.863‐6.060)	.001	3.088 (1.645‐5.796)
Targeted therapy	.002	2.014 (1.289‐3.147)			.696	0.889 (0.491‐1.607)		
Pathological response	.566	0.873 (0.548‐1.389)			.055	0.524 (0.271‐1.013)		
Clinical response	.098	0.687 (0.441‐1.071)			.002	0.392 (0.215‐0.715)		
Postoperative chemotherapy	.083	0.679 (0.438‐1.052)			.012	0.475 (0.265‐0.849)		

Abbreviations: BMI, body mass index; CI, confidence interval; CRLM, colorectal cancer liver metastasis; HR, hazards ratio; NAC, neoadjuvant chemotherapy; OS, overall survival; PFS, progression‐free survival; TTS, time to surgery.

#### 3.6.2 After 1:2 propensity matching

**FIGURE 5:**
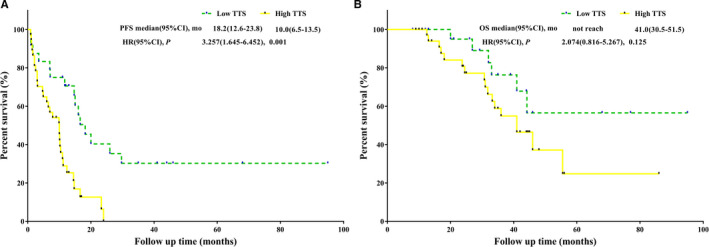
After propensity matching. A, PFS analysis of high TTS vs low TTS. B, OS analysis of high TTS vs low TTS. OS, overall survival; PFS, progression‐free survival; TTS, time to surgery

The median follow‐up time was 42 months. Forty‐six patients (75.41%) experienced recurrence, and 23 patients (37.70%) died. The median OS and the median PFS were 44 months (95% CI 35.1‐53.3) and 11.2 months (95% CI 8.6‐13.8), respectively. Compared to patients with a high TTS, patients with a low TTS held significantly better PFS (*P *<* *.001, mPFS: 18.2 months vs 10 months) and an equivalent OS (*P* = .115, mOS: not reached vs 41 months) (Figure 5).

Major liver resection, TTS > 5 weeks, >7 NAC cycles, targeted therapy, and postoperative chemotherapy were associated with decreased PFS in univariate analysis. Multivariate analysis showed that TTS > 5 weeks (HR = 3.031, 95% CI: 1.494‐6.149, *P* = .002) and >7 NAC cycles (HR = 4.478, 95% CI: 1.719‐11.667, *P* = .002) were independent predictors of decreased PFS (Table 4). TTS was not a significant predictor of OS in univariate analysis and multivariate analysis (Table 4).

**TABLE 4:** Univariate and multivariate analyses of predictive factors of PFS and OS for CRLM patients after 1:2 propensity matching

Factor	PFS				OS			
	Univariate analysis		Multivariate analysis		Univariate analysis		Multivariate analysis	
	Value *P*	HR (95% CI)	Value *P*	HR (95% CI)	Value *P*	HR (95% CI)	Value *P*	HR (95% CI)
Age > 60 y	.082	0.577 (0.310‐1.073)			.244	0.587 (0.239‐1.439)		
Male	.625	1.163 (0.635‐2.130)			.867	1.076 (0.454‐2.550)		
Preoperative CEA > 10 ng/mL	.614	1.162 (0.648‐2.086)			.966	0.982 (0.433‐2.230)		
BMI > 24 kg/m^2^	.809	1.075 (0.599‐1.931)			.538	1.299 (0.565‐2.988)		
ALB > 40 g/L	.875	0.952 (0.517‐1.752)			.420	1.443 (0.592‐3.517)		
Comorbidity	.476	1.240 (0.686‐2.239)			.757	0.877 (0.382‐2.012)		
ASA score 3‐4	.857	1.099 (0.393‐3.072)			.787	0.845 (0.248‐2.873)		
High CONUT score	.112	1.621 (0.894‐2.942)			.103	2.001 (0.870‐4.602)		
Synchronous metastasis	.524	0.778 (0.359‐1.684)			.001	0.187 (0.069‐0.507)		
Left hemicolon	.405	1.488 (0.584‐3.789)			.471	0.478 (0.064‐3.558)		
R0 resection	.180	0.669 (0.371‐1.204)			.502	0.733 (0.295‐1.817)		
Major liver resection	.028	1.995 (1.079‐3.687)			.492	1.342 (0.579‐3.111)		
Bilobar distribution	.233	1.423 (0.797‐2.540)			.969	0.984 (0.429‐2.256)		
Extrahepatic metastases	.509	1.314 (0.585‐2.952)			.536	1.471 (0.433‐4.998)		
Diameter of metastases ≥ 3 cm	.668	1.139 (0.629‐2.061)			.485	1.346 (0.585‐3.095)		
Multiple metastases	.281	1.414 (0.753‐2.655)			.830	0.910 (0.385‐2.150)		
Complications	.996	1.001 (0.561‐1.789)			.517	1.314 (0.575‐3.000)		
Minor complications	.822	1.074 (0.578‐1.995)			.352	1.480 (0.648‐3.381)		
Major complications	.804	0.911 (0.439‐1.893)			.694	0.744 (0.171‐3.234)		
TTS > 5 wk	.001	3.257 (1.645‐6.452)	.002	3.031 (1.494‐6.149)	.125	2.074 (0.816‐5.267)		
Poor differentiation	.301	1.394 (0.743‐2.613)			.432	1.434 (0.584‐3.522)		
pT3‐T4	.148	1.988 (0.784‐5.039)			.247	2.365 (0.551‐10.163)		
Node‐positive primary tumor	.100	1.739 (0.899‐3.366)			.548	1.317 (0.537‐3.233)		
NAC toxicity	.549	1.280 (0.571‐2.867)			.301	2.885 (0.388‐21.463)		
Neutropenia	.685	1.128 (0.631‐2.016)			.989	1.006 (0.442‐2.286)		
Heterochronous resection	.068	0.549 (0.289‐1.045)			.001	4.225 (1.835‐9.729)	.004	3.653 (1.526‐8.745)
Preoperative chemotherapy								
Oxaliplatin‐based regimen	.057	0.447 (0.195‐1.023)			.002	0.195 (0.069‐0.552)	.022	0.268 (0.087‐0.825)
Cycles > 7	<.001	7.449 (2.924‐18.979)	.002	4.478 (1.719‐11.667)	<.001	3.360 (1.863‐6.060)		
Targeted therapy	.002	2.603 (1.439‐4.710)	.005	2.398 (1.295‐4.439)	.197	0.550 (0.221‐1.364)		
Pathological response	.916	0.969 (0.539‐1.741)			.021	0.357 (0.149‐0.857)		
Clinical response	.449	0.792 (0.434‐1.448)			.068	0.435 (0.178‐1.063)		
Postoperative chemotherapy	.024	0.510 (0.284‐0.951)			.128	0.525 (0.229‐1.203)		

Abbreviations: BMI, body mass index; CI, confidence interval; CRLM, colorectal cancer liver metastasis; HR, hazards ratio; NAC, neoadjuvant chemotherapy; OS, overall survival; PFS, progression‐free survival; TTS, time to surgery.

## 4 DISCUSSION

This study implemented X‐tile analyses to objectively identify the optimal timing of resection for CRLM after NAC. Five weeks from the completion of NAC to liver resection for CRLM is an inflection point in pathological response and survival. The cohort of patients receiving resection between 4 and 5 weeks after NAC demonstrated a higher rate of favorable pathological response and better PFS. A 1:2 propensity score matching analysis confirmed this finding. Thus, 5 weeks represent an adverse inflection for resection with unfavorable outcomes. To our knowledge, this study is the first to investigate such a finding in CRLM patients.

The TTS for CRLM is an important question without a definite conclusion frequently confused by patients and surgeons. The current clinical guidelines^6^, ^7^ recommend that the resection for CRLM is usually scheduled after 4 weeks from the last dose of NAC. However, there has not been a clinical trial designed to define the specific optimal timing of surgery after NAC for CRLM patients. Clinicians have to rely on clinical experiences or extrapolate from interval data from adjuvant therapy. However, the applicability of that in preoperative settings has not been validated.

Previous studies have revealed that a prolonged TTS significantly increased the odds of pCR for esophageal and rectal patients with nCRT^8^, ^9^, ^11^, ^12^ and gastric cancer patients with NAC.^10^ Another study showed that a longer TTS from the end of nCRT to surgery did not increase the rate of pCR in esophageal cancer.^14^ In contrast, we found that a longer interval was associated with a higher rate of unfavorable pathological responses. The possible reasons are as follows: (a) The pathological response is the result of the interaction of neoadjuvant therapy and tumor progression.^27^ The treatment strategy in previous studies^8^, ^9^, ^11^, ^12^, ^14^ was nCRT, which had a strong delayed effect. A higher rate of pCR could be obtained by prolonging the TTS. However, the patients in this study received NAC, and NAC has not been confirmed to have a delayed effect. (b) The esophageal and rectal cancer patients in previous studies^8^, ^9^, ^11^, ^12^, ^14^ were not associated with distant metastasis. The subjects included in this study were colorectal cancer patients with distant metastasis, which may be more advanced with worse biological behaviors. The extension of the TTS may enhance the possibility of CRLM progression. (c) The research focuses are different. The focus of previous studies was the impact of the TTS on pCR. Our study investigated the relationship between the TTS and favorable TRG, which was not clear in previous studies. Investigating whether prolonged TTS increased the odds of pCR was not allowed due to the inferior chemosensitivity of CRLM; thus, there were very few cases of complete pathological response in this study.

This study revealed that patients with a high TTS were more likely to have unfavorable PFS, which was consistent with previous studies suggesting that postponing TTS impairs survival in many cancers, such as esophageal and ovarian cancers.^13^, ^14^ In esophageal cancer patients, worse perioperative mortality, and OS are significantly correlated with a longer time interval between nCRT and surgery.^14^ Ming Chen et al^13^ analyzed the data from an ovarian cancer patient cohort treated with NAC, revealing a detrimental effect of a TTS > 4 weeks on PFS and no relationship between the TTS and OS. However, these studies determined the time intervals arbitrarily and did not provide better resolution in the ranges chosen. On the contrary, we utilized a novel statistical method with an unbiased way to investigate an inflection point in clinical outcomes. Why the high TTS was associated with unfavorable PFS is relevant from several perspectives: the longer TTS may intensify the therapy‐induced fibrotic changes and local inflammation,^28^ and this time period may be theoretically correlated with tumor regrowth, which may make surgical resection more difficult, thereby worsening surgical outcomes. In addition, shrinkage of the primary tumor can stimulate residual tumor growth, which has been investigated in animal models.^29^ Therefore, unnecessary extension in the TTS for CRLM patients might be avoided. The operation should be performed at the earliest opportunity after recovery from NAC.

The equivalent OS between a high TTS group and a low TTS group can be explained as follows: First, palliative treatments, which are thought to prolong survival after recurrence, were not considered in this study because of inadequate data. Differences in whether patients received palliative treatments and palliative treatment strategies between the high group and low TTS group weaken the survival advantage of the low TTS group. Second, the OS in this study was defined as the date of surgery to the date of death caused by cancer‐related or noncancer‐related reasons. Noncancer‐related deaths may weaken the prognostic influence of the TTS.

The results of this study revealed that favorable TRG has no effect on prognosis, which can be explained as follows: Some studies have shown that patients with complete pathological response significantly improved survival after resection in gastrointestinal cancer and rectal cancer.^30^, ^31^, ^32^ The effect of a partial pathological response on survival is less clear. However, the results of some studies revealed that a partial pathological response did not improve the prognosis than a nonpartial pathological response.^32^, ^33^ In this study, the favorable TRG (TRG 1‐3) was defined as complete or partial pathological responses. The size of patients with complete pathological response in this study was very small. The interference from the partial pathological response may weaken the prognostic influence of the complete pathological response. The impact of complete pathological response on prognosis was not investigated because of the limited cases of complete responders in the current single cohort.

There were several factors leading to the delayed operation. Our study demonstrated that patients with a high TTS were more likely to receive NAC > 7 cycles (29.7% vs 7.4%). The more NAC cycles was an important factor for the delayed operation, the reasons of which may be that the more NAC cycles was an independent predictor for poor performance status after NAC and the poor performance status needed more TTS to recovery.^34^, ^35^ Our study also revealed that NAC > 7 cycles was an independent predictor for the survival. The propensity score matching in this study was performed between high TTS patients and low TTS patients to eliminate the bias from the impact of this factor on survival. In addition, other studies suggested the most common factors for delayed operation included heavily economic level of patients, the management of health insurance, and the busy turnover of beds in hospital.^36^, ^37^

There were some limitations in our study. First, this was a retrospective investigation based on a single institution experience. Second, when investigating the causes of delayed operation, this study failed to monitor the nutritional status during and after NAC, so it failed to confirm the correlation between NAC cycles and nutritional status in CRLM patients. In addition, the study failed to include factors such as economic level of patients and the management of health insurance in the analysis because of the inadequate data. Third, this study held the selection bias, given that the TTS was determined due to multiple clinical factors, which demonstrated that the TTS was chosen based on the clinical condition and not on randomized regulations. The biases of these factors were adjusted by the multivariable analysis and propensity score matching, but the lack of randomization could still influence our results. Therefore, our results need to be validated by a multicenter randomized control trial.

In conclusion, our findings suggest that the longer interval between NAC and surgery may be disadvantageous for a favorable pathological response and long‐term PFS. These results should be validated prospectively in a randomized trial.

## CONFLICT OF INTEREST

The authors have declared that no conflict of interest exists.

## AUTHOR CONTRIBUTION

Jianguo Zhou and Hong Zhao were involved in conception and design. Jianguo Zhou, Hong Zhao, and Jianqiang Cai were involved in administrative support. Qichen Chen and Rui Mao were involved in provision of study materials of patients and data analysis and interpretation. All authors were involved in collection and assembly of data, manuscript writing, and final approval of manuscript.

## Data Availability

The datasets generated during the current study are available from the corresponding author on reasonable request.
